# Root biomass and root traits of *Alnus glutinosa* show size-dependent and opposite patterns in a drained and a rewetted forest peatland

**DOI:** 10.1093/aob/mcaa195

**Published:** 2020-09-10

**Authors:** Sarah Schwieger, Gesche Blume-Werry, Felix Ciesiolka, Alba Anadon-Rosell

**Affiliations:** 1 Experimental Plant Ecology, Institute of Botany and Landscape Ecology, Greifswald University, Greifswald, Germany; 2 Landscape Ecology and Ecosystem Dynamics, Institute of Botany and Landscape Ecology, Greifswald University, Greifswald, Germany

**Keywords:** Alder forest, *Alnus glutinosa*, annual growth rings, biomass distribution, fine roots, forest peatland, functional traits, root age, rewetting, specific root area

## Abstract

**Background and Aims:**

Forest peatlands represent 25 % of global peatlands and store large amounts of carbon (C) as peat. Traditionally they have been drained in order to increase forestry yield, which may cause large losses of C from the peat. Rewetting aims to stop these losses and to restore the initial storage function of the peatlands. As roots represent major peat-forming elements in these systems, we sampled roots with diameter <5 mm in a drained and a rewetted forest peatland in north-east Germany to evaluate differences in tree biomass investments below ground, root functional characteristics and root age.

**Methods:**

We cored soil next to *Alnus glutinosa* stems and sorted root biomass into <1, 1–2 and 2–5 mm diameter classes. We measured biomass distribution and specific root area (SRA) in 10-cm depth increments down to 50 cm, and estimated root age from annual growth rings.

**Key Results:**

Root biomass in the rewetted site was more than double that in the drained site. This difference was mostly driven by very fine roots <1 mm, which accounted for 51 % of the total root biomass and were mostly (75 %) located in the upper 20 cm. For roots <1 mm, SRA did not differ between the sites. However, SRA of the 1–2 mm and 2–5 mm diameter roots was higher in the drained than in the rewetted site. Root age did not differ between sites.

**Conclusions:**

The size-dependent opposite patterns between root biomass and their functional characteristics under contrasting water regimes indicate differences between fine and coarse roots in their response to environmental changes. Root age distribution points to similar root turnover rates between the sites, while higher root biomass in the rewetted site clearly indicates larger tree C stocks below ground under rewetting, supporting the C sink function of the ecosystem.

## INTRODUCTION

Forest peatland ecosystems account for up to 25 % of peatlands globally. They play a major role in the global carbon (C) cycle by sequestering high amounts of C as plant biomass and through long-term storage of C as peat (Zoltai and Martikainen, 1996). Forest peatlands are dominated by tree species that tolerate permanent to semi-permanent flooding. Across Europe, black alder (*Alnus glutinosa*) is a widespread species, typically found in mixed broadleaved forests in wet areas (Claessens *et al.*, 2010) and known to be peat-forming (Succow and Joosten, 2001; Barthelmes *et al.*, 2010; Jurasinski *et al.*, 2020). Drainage of peatlands and subsequent use for agriculture or forestry have strongly reduced the occurrence of wet alder forest peatlands (Prieditis, 1997). These areas have effectively been turned into C sources through enhanced decomposition of peat, reversing their climate change mitigation function (Joosten *et al.*, 2016; Leifeld *et al.*, 2019). In an effort to counteract these consequences, more and more sites are being rewetted, but so far it is unclear how rewetting will affect C cycling in these forest peatlands and, ultimately, their soil C storage function.

Roots are pivotal elements in soil C cycling and storage, as they constitute up to 60 % of net primary production in forest ecosystems (Jackson *et al.*, 1997). In fact, in many peatland ecosystems roots are key peat-forming elements (Succow and Joosten, 2001; Jurasinski *et al.*, 2020). With their short lifespan, high nutrient content and high respiration rates, fine roots (≤2 mm in diameter) are more important in terms of C and nutrient budgets than larger-diameter woody roots (Iversen *et al.*, 2015).

Plants respond to environmental changes through plastic adjustments of their traits. Functional traits are a useful tool for studying how such changes affect ecosystem processes (Bardgett *et al.*, 2014; Díaz *et al.*, 2016). Most trait-related research has focused on above-ground plant traits, but it is increasingly recognized that root traits are pivotal for ecosystem processes like C and nutrient cycles (Iversen *et al.*, 2015; McCormack *et al.*, 2015). However, our understanding of the response of root functional traits to environmental changes and their link to root growth and lifespan is still very limited. Thus, research on root traits is necessary to understand plant adjustments to rewetting and their potential effects on soil C storage.

There are several root traits that may be important for plant functioning and C storage. For example, biomass distribution of fine roots, specific root area (SRA; root surface area per mass) and specific root length (SRL) influence the rates of resource uptake (Jackson *et al.*, 1997; Makita *et al.*, 2012; McCormack *et al*., 2012; Bardgett *et al.*, 2014). Specific root area is tightly linked to root functioning, since roots with a greater surface area per biomass are able to explore larger soil volumes more efficiently (Hajek *et al.*, 2013). Biomass distribution of roots throughout the soil profile is related to soil properties, as these change with soil depth (e.g. oxygen content, soil moisture, bulk density, temperature and soil texture) (Schenk and Jackson, 2005). Similarly, root traits may differ with depth, affecting nutrient uptake and C storage.

Root age estimations provide valuable information about root turnover and, therefore, the inputs of C from roots into the soil and the persistence of organic matter as living roots before they are decomposed and converted to soil organic matter (Eissenstat and Yanai, 1997). Still, root age is amongst the most overlooked parameters in forest ecosystem research. Direct observation of fine-root longevity done *in situ* with minirhizotrons (e.g. Withington *et al.*, 2006; McCormack *et al.*, 2013) seems to systematically overestimate fine-root age and turnover (Strand *et al.*, 2008), because it favours the sampling of smaller and more dynamic lower-order roots (Guo *et al.*, 2008). The determination of fine-root age from the analysis of C isotopes (e.g. Gaudinski *et al.*, 2001; Sah *et al.*, 2011), however, tends to systematically underestimate the turnover of individual roots (Strand *et al.*, 2008). The reasons for such underestimation are the time lag between C acquisition and the use of that same C for fine-root growth, and also the uptake of older C sources from the soil for growing new roots (Solly *et al.*, 2018). Root age determination with annual growth rings is a less common approach, although it has been successfully applied in tree species from seasonal climates (Vurdu, 1977; Reynolds, 1983; Solly *et al.*, 2018). This approach does not require repeated sampling over years, as does the minirhizotron method, but still informs about the age of the sampled roots (and not the age of the C used, as C isotopes do).

Understanding patterns in the distribution and functioning of fine-root biomass and linkages to the water regime in forested peatland ecosystems will improve our ability to predict ecosystem responses to environmental changes, and the consequences of restoration through rewetting of formerly drained peatlands. Here, we asked whether the water regime (drained or rewetted) of a temperate peatland forest affects (1) (fine) root biomass and (2) (fine) root functional characteristics (i.e. SRA) throughout the depth profile, and (3) (fine) root age. Therefore, we sampled roots of *A. glutinosa*, sorted them into two fine-root classes (<1 mm and 1–2 mm) and one coarse root diameter class (2–5 mm) and measured biomass distribution and SRA down to 50 cm, as well as root age by counting annual growth rings. Despite not having information on root production before the rewetting, we here provide an assessment of below-ground biomass and root functional traits for these two forests, each one under a different water regime.

## MATERIALS AND METHODS

### Study site

The study sites are located in Wöpkendorf, in north-eastern Germany. The study region has a maritime climate with a mean annual temperature of 8.8 °C and mean annual precipitation of 601 mm. Typically, January is the coldest month, with a mean temperature of 0.7 °C and a mean precipitation of 46 mm, while July is the warmest month, with a mean temperature of 17.8 °C and a mean precipitation of 59 mm (meteorological data provided by Germany’s National Meteorological Service from 1981 to 2010).

The study sites are a drained (54°08′06″ N, 12°32′11″ E, 44 m a.s.l., ~0.27 ha) and a rewetted alder stand (54°07′37″ N, 12°29′04″ E, 37 m a.s.l., ~0.75 ha) within the same forest, which was originally a natural alder forest. This alder forest is a deciduous fen woodland, dominated by *Alnus glutinosa* with a poorly developed shrub layer, but a species-rich field layer. These kinds of forest prefer base-rich soil conditions, often-moving groundwater and a rather constant water table (Joosten *et al.*, 2017). Both sites were drained for wood pasture at the end of the 18th century. In 2003, a rewetting action was initiated in the rewetted stand (Bönsel, 2006) by filling the ditches to stop the drainage. The vegetation of the drained alder stand is characterized by a mixed stand of *A. glutinosa* and a few *Fraxinus excelsior* individuals. The understorey is dominated by *Urtica dioica* (~45 % cover) and *Rubus idaeus* (~40 % cover). The peat layer in the drained site is rather shallow (~60 cm) and strongly degraded. In the rewetted alder stand, *A. glutinosa* is the only tree species and its understorey is dominated by *Carex riparia* (~70 % cover) with *Glyceria fluitans* (~30 % cover) and *Solanum dulcamara* (~20 % cover). The peat layer in the rewetted site reaches >2 m in depth.

Both sites are study sites within the interdisciplinary joint project WETSCAPES, aiming to develop scientific principles for sustainable cultivation of wet peatlands, particularly of drained areas that were later rewetted (Jurasinski *et al.*, 2020). Our study compared two sites with replicates within each site but not for each treatment (i.e. water regime) because appropriate, additional replicates at the peatland forest type level are not readily available in this region, in particular for the rewetted state. Nonetheless, the selected forest stands are representative of the peatland forest types present in the area (Jurasinski *et al.*, 2020).

### Study species

*Alnus glutinosa* is a typical species of mixed broadleaved forest on wet sites and is widespread across Europe, representing ~5 % of the forest area in Central Europe (Claessens *et al.*, 2010). The root system of *A. glutinosa* is very well adapted to water-saturated conditions; it tolerates stagnant waters (McVean, 1956) and trees are therefore often found on peatland sites (Succow and Joosten, 2001). *Alnus glutinosa* is an important pioneer species that is able to fix nitrogen (N) through symbiotic interactions with the bacterium *Frankia alni*, as well as with ecto- and arbuscular mycorrhizal fungi (McVean, 1953; Bond *et al.*, 1954). Through these effective symbioses, *A. glutinosa* can facilitate nutrient uptake in N-poor conditions (Barea and Azcon-Aguilar, 1983; Orfanoudakis *et al.*, 2010).

### Root sampling

At each study site, five soil cores (ø = 18.8 cm, length = 50 cm) were taken between November 2018 and February 2019, each at 80 cm distance from an individual tree. The soil was pre-cored with a metal pipe corer (Clymo, 1988), and a saw was used to cut large roots to avoid soil compression with the corer insertion. Afterwards, the soil cores were extracted with a plastic (PVC) pipe and sealed with a lid. Cores were stored at +4 °C until processing. We divided the cores into five slices spanning 10 cm depth intervals (0–10, 10–20, 20–30, 30–40 and 40–50 cm). Undecomposed roots were washed, sieved with meshes of 1, 2 and 5 mm and assigned to three diameter classes: very fine roots of <1 mm, fine roots of 1–2 mm and coarse roots of 2–5 mm. Large structural roots were avoided with our sampling method. Roots with a diameter >5 mm found in our cores were discarded in further analysis. We oven-dried the root material at 60 °C for 48 h, and weighed it to calculate dry weight fine-root biomass for every depth and diameter class.

To avoid unwanted effects of stand density differences between the sites (rewetted, ~311 trees per ha; drained, ~273 trees per ha) on our root biomass measurements, we sampled in close proximity (80 cm) of individual tree stems so that our biomass estimates represent the cored individual tree. We tried to minimize differences in tree size (i.e. diameter at breast height and height), but also in microtopography (avoiding hollows and hummocks) (see data on tree metrics in Supplementary Data Table S1).

### Determination of SRA

We selected a subsample of each diameter class and depth to estimate the root area. We used the program IJ_Rhizo (Pierret *et al.*, 2013), written as a macro for ImageJ (Abràmoff *et al.*, 2004).

We calculated SRA by using the ratio of acquisition (i.e. *A* = root area in cm^2^) to resource investment (i.e. *M* = mass in g):

$$\begin{array}{l} SRA=\frac{\;A\;}{M} \end{array}$$

### Root age determination

**Fig. 1.**
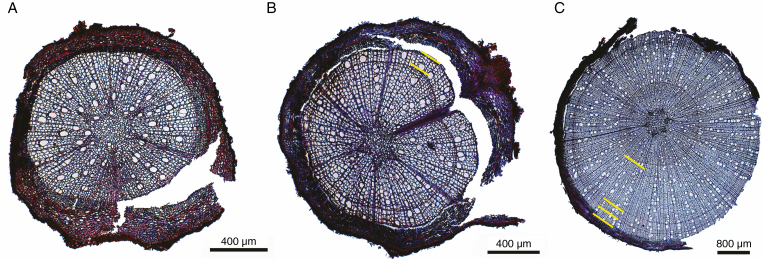
Cross-sections of *A. glutinosa* roots. (A) A <1-year-old root, (B) a 2-year-old root and (C) a 4-year-old root. Yellow lines indicate the annual growth ring boundaries. Sections (A) and (B) belong to the rewetted stand and section (C) belongs to the drained stand.

We selected three roots per diameter class (<1, 1–2 and 2–5 mm) within the 10- to 20-cm depth slices for root age analyses (*N* = 90). We prepared 15-µm-thick root cross-sections with a rotary microtome (Leica RM 2245, Leica Microsystems, Germany), which were stained with a 1:1 mixture of safranin and astra blue, rinsed with ethanol solutions of increasing concentration (50 %, 70 %, 96 %), embedded in Euparal and dried at 65–70 °C for 48 h. We photographed the sections at ×50 or ×100 magnification with a digital camera attached to a microscope (Leica DM 2500, Leica Microsystems, Germany). For the larger root sections, we took several 30 % overlap photographs per section and stitched them with the open-source software ImageJ (Abràmoff *et al.*, 2004; Preibisch *et al.*, 2009; Schindelin *et al.*, 2012). For each section, we counted the annual growth rings and measured root diameter with Image J (Fig. 1).

### Abiotic parameters and nutrient availability

General weather data were recorded at a local weather station ~1 km from either study site.

**Fig. 2.**
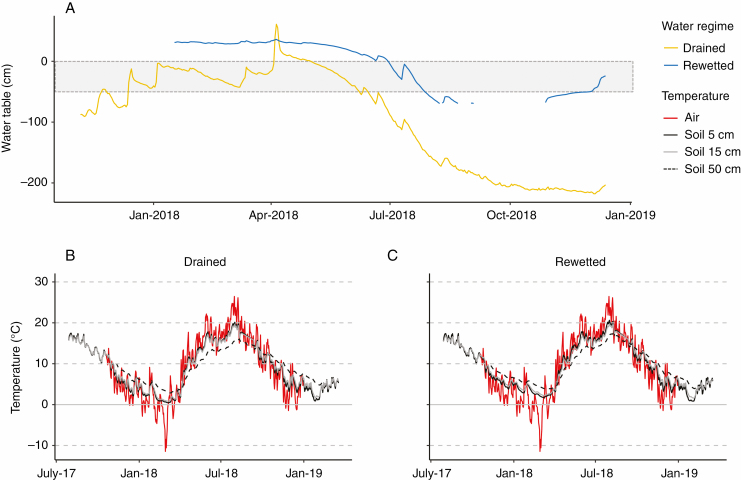
(A) Groundwater table (cm) for the drained and rewetted sites. The grey ribbon indicates the area where sampled roots were located. Gaps in water table data between 23 August and 27 October 2018 resulted from water tables below 70 cm, which exceeded the groundwater pipe in this site. (B, C) Air temperature, and soil temperature at depths of 5, 15 and 50 cm for the (B) drained and (C) rewetted site.

The groundwater table relative to the soil surface was recorded at 15-min intervals in a slotted PVC pipe using a CS456 pressure transducer connected via an SDI-12 sensor to a CR1000 data logger (Campbell Scientific, Bremen, Germany) at each site (Fig. 2A). Gaps in water table data recording between 23 August and 27 October 2018 resulted from water tables below 70 cm, which exceeded the reach of the groundwater pipe at this site.

Soil water content was measured by taking 4–5 g soil samples at three depths (0–5, 15–20 and 25–30 cm) in three locations at each study site. Sampling started in April 2017 and was performed every 4 months in 2017 and every 2 months in 2018 (Supplementary Data Fig. S1). Soil samples were incubated for 24 h in a drying chamber at 90 °C to remove the water. Gravimetric water content (i.e. mass of water per mass of dry soil) was calculated as the percentage weight decrease after drying.

Soil temperature data were collected using Hobo data loggers (Onset Computer Corporation, Bourne, MA, USA) at 15-min intervals at 5 and 15 cm depth by six loggers per site. Air temperature was recorded at each site at 2 m height using a CR300 data logger (Campbell Scientific, Bremen, Germany) and averaged over 30 min (Fig. 2B, C).

**Fig. 3.**
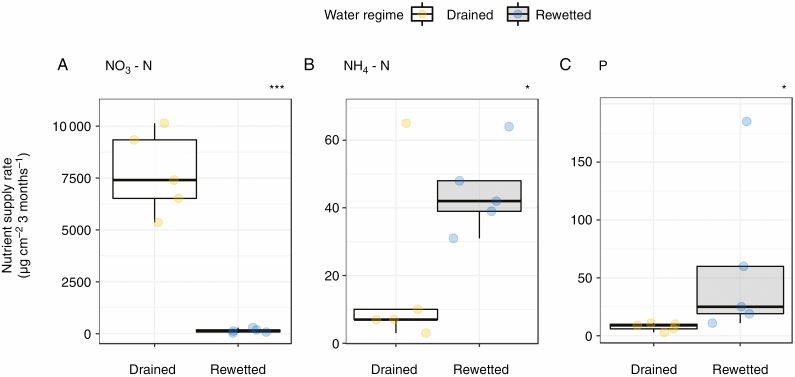
Nutrient supply rate (µg cm^−2^ 3 months^−1^) for (A) nitrate (NO_3_-N), (B) ammonium (NH_4_-N) and (C) plant-available P for the drained and rewetted sites estimated with PRS™ probes between 7 July and 18 October 2018. Asterisks indicate significant differences between the two water regimes (drained and rewetted) across the depth profile (*P < 0.05, ***P < 0.001).

Between 7 July and 18 October 2018 we measured nutrient supply rate (µg cm^−2^ burial time^−1^) for nitrate (NO_3_-N), ammonium (NH_4_-N) and phosphorus (P) available to plants using Plant Root Simulator (PRS)™ probes with ion-exchange membranes (Western Ag Innovations, Saskatoon, SK, Canada; Fig. 3). Two pairs of anion and cation PRS™ probes (total surface area = 17.5 cm^2^) were inserted vertically for 10 cm into the soil in five plots at each site and then pooled per plot for analysis (drained, *n* = 5; rewetted, *n* = 5). After removal, the PRS™ probes were washed in the laboratory with deionized water, and were thoroughly scrubbed with a coarse brush to ensure complete removal of residual soil. Afterwards, the probes were send to the Western Ag laboratory for analysis. For a detailed description of the methods used for the analysis of the PRS™ probes see Hangs *et al.* (2004).

### Data analysis

All statistical analyses and visualizations were done in R version 3.5.3 (R Core Team, 2019). We tested for differences in root biomass, SRA and age between water regime (drained and rewetted), root diameter class (<1, 1–2 and 2–5 mm), depth (0–10, 10–20, 20–30, 30–40 and 40–50 cm) and their interactions with a linear mixed-effect model ANOVA [R package lmerTest, version 3.1–0; Kuznetsova *et al.* (2017); and nlme, version 3.1–137; Pinheiro *et al.* (2018)]. We used water regime, diameter class and depth as interacting explanatory variables, and core ID as a random factor (i.e. identity of the sampled tree individual per site). Models were fitted with the restricted maximum likelihood (REML) estimation method. For each model, we plotted the residuals with normal Q–Q plots and residual versus fitted values to graphically test the assumptions of normality and homogeneity of variance. When necessary, data were log- or square root-transformed to meet the assumptions. We used the varIdent structure (Zuur *et al.*, 2009) when the homogeneity of residuals was not reached. A Tukey’s HSD test [R package emmeans, version 1.4.1; Lenth (2019)] was used for each response variable to test for differences within water regimes, depths and diameter classes. We tested whether root age can be predicted by diameter size with a simple linear regression. Visualizations were done using the package ggplot2 [version 3.2.1; Wickham (2016)]. Effects were considered significant at *P* < 0.05.

## RESULTS

### Site characteristics

The two studied alder forest stands are in close proximity to each other and therefore share a macroclimate but differ in their microclimate (Supplementary Data Table S2). Mean annual air temperature was 9.9 °C, while soil temperatures varied between 9.4 and 10.2 °C (Fig. 2B, C). In 2018, water tables dropped down below −70 cm at both sites (Fig. 2A). Still, the rewetted sites experienced water saturation close to or exceeding the soil surface ~40 % more time during 2018 than the drained site (Supplementary Data Table S2). Similarly, soil water content was significantly higher in the rewetted site for all measured depths (0–5, 15–20 and 25–30 cm) compared with the drained site (rewetted, 77 ± 0.79 %; drained, 46 ± 0.86 %; *F* = 718.7, *P* < 0.001; Supplementary Data Fig. S1). There was no significant water regime × depth interaction (*F* = 0.2, *P* = 0.79).

The amount of NO_3_-N was 50 times higher in the drained site (7755 ± 883 µg cm^−2^ 3 months^−1^) than in the rewetted site (145 ± 45 µg cm^−2^ 3 months^−1^; *F* = 197.7, *P* < 0.001; Fig. 3A). We found almost 2.5 times higher availability of NH_4_-N (rewetted, 45 ± 6 µg cm^−2^ 3 months^−1^; drained, 18 ± 12 µg cm^−2^ 3 months^−1^; *F* = 8.0, *P* = 0.022; Fig. 3B) and ~7.5 times higher availability of P (rewetted, 6.00 ± 3.24 µg cm^−2^ 3 months^−1^; drained, 0.78 ± 0.15 μg cm^−2^ 3 months^−1^; *F* = 8.7, *P* = 0.018; Fig. 3C) in the rewetted site compared with the drained site.

### Root biomass

**Fig. 4.**
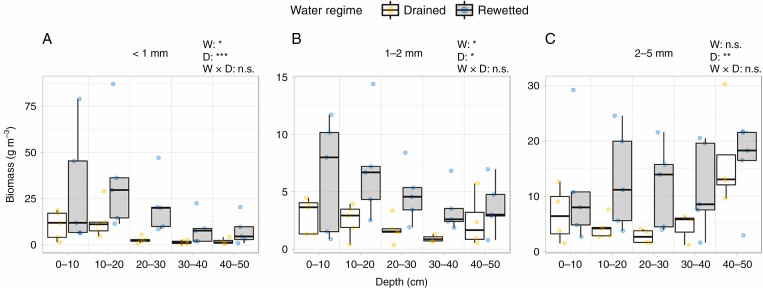
Root biomass distribution (g dry weight m^−3^) across the depth profile (0–10, 10–20, 20–30, 30–40 and 40–50 cm) for the diameter classes (A) <1 mm, (B) 1–2 mm and (C) 2–5 mm in the drained and rewetted sites. Asterisks indicate significant differences between fixed-effect variables (n.s., *P* > 0.05; *P < 0.05, **P < 0.01, ***P < 0.001). W, water regime; D, depth.

Water regime had a significant effect on total root biomass (≤5 mm), which was higher in the rewetted site (192.4 ± 55.9 g m^−3^, mean ± s.e.) than in the drained site (67.0 ± 11.7 g m^−3^) (*F* = 8.1, *P* = 0.022). However, the significant water regime × diameter class interaction (*F* = 2.6, *P* = 0.079) indicated that root biomass was significantly higher under rewetting for the <1 mm (Tukey HSD, *P* = 0.005) and 1–2 mm classes (Tukey HSD, *P* = 0.036), but not for the 2–5 mm class (Tukey HSD, *P* = 0.085). Diameter class also had a significant effect on root biomass (*F* = 29.1, *P* < 0.001). Roots belonging to the <1 mm diameter class had the highest biomass and made up 51 % of the total root biomass sampled. Root biomass of the 1–2 mm diameter class was the lowest and accounted only for 14 % of the total root biomass. Biomass of the <1 mm fine roots (rewetted, 103.4 ± 41.0 g m^−3^; drained, 29.5 ± 5.1 g m^−3^) was 3 times as high as the 1–2 mm fine roots (rewetted, 25.3 ± 6.7 g m^−3^; drained, 9.8 ± 2.4 g m^−3^; Tukey HSD, rewetted *P* < 0.001, drained *P* = 0.004), but showed no significant difference from the 2–5 mm roots (rewetted, 63.7 ± 14.0 g m^−3^; drained, 27.7 ± 7.0 g m^−3^). Root biomass differed significantly with depth (*F* = 9.6; *P* < 0.001), but we also found a significant water regime × depth (*F* = 2.9, *P* = 0.025) and a diameter class × depth (*F* = 5.5, *P* < 0.001) interaction. Root biomass was higher at the rewetted site than at the drained site from soil surface to 40 cm depth, but not at 40–50 cm, where there were no clear differences between water regimes (Tukey HSD, *P* = 0.209; Fig. 4). Root biomass of the diameter class <1 mm was higher in shallow depths and declined with increasing depth (Fig. 4A), while roots of the 1–2 mm class showed no clear difference along the soil profile (Fig. 4B), and roots of 2–5 mm diameter slightly increased with increasing depth (Fig. 4C; Supplementary Data Table S3).

### Specific root area

**Fig. 5.**
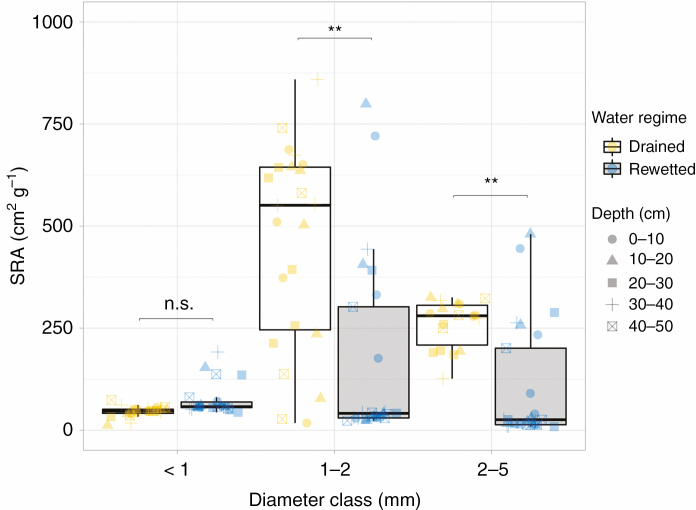
SRA (cm^2^ g^−1^ dry weight) of roots of the diameter classes <1, 1–2 and 2–5 mm at soil depths 0–10, 10–20, 20–30, 30–40 and 40–50 cm in the drained and rewetted sites. **P < 0.01, significant difference between the two water regimes (drained and rewetted) across the depth profile; n.s., *P* > 0.05.

Diameter class had a significant effect on SRA (*F* = 110.1, *P* < 0.001). Roots of the diameter class 1–2 mm had the highest SRA (drained, 460 ± 51 cm^2^ g^−1^; rewetted, 178 ± 49 cm^2^ g^−1^), followed by the 2–5 mm roots (drained, 260 ± 14 cm^2^ g^−1^; rewetted, 97 ± 27 cm^2^ g^−1^) and the <1 mm roots with the lowest SRA (drained, 46 ± 3 cm^2^ g^−1^; rewetted, 75 ± 9 cm^2^ g^−1^). Water regime had a significant effect on SRA, which was higher in the drained site than in the rewetted site (*F* = 21.5, *P* = 0.002; Fig. 5). However, the significant water regime × diameter class interaction (*F* = 37.7, *P* < 0.001) showed that the drained site had significantly higher values for the 1–2 and 2–5 mm diameter classes (Tukey HSD, 1–2 mm *P* = 0.007, 2–5 mm; *P* = 0.001), but not for the <1 mm roots (in this case there was only a trend towards larger values under rewetting; Tukey HSD, <1 mm, *P* = 0.062). The SRA of the diameter class <1 mm was significantly lower than that of the diameter classes 1–2 mm (Tukey HSD, *P* < 0.001) and 2–5 mm (Tukey HSD, *P* < 0.001) in the drained site, while the SRA in the rewetted site did not differ between diameter classes. Soil depth had a significant effect on SRA (*F* = 3.3, *P* = 0.013, Supplementary Data Fig. S2). SRA was highest shallow soil layers (0–10 cm) and decreased with increasing depth.

### Root age

**Fig. 6.**
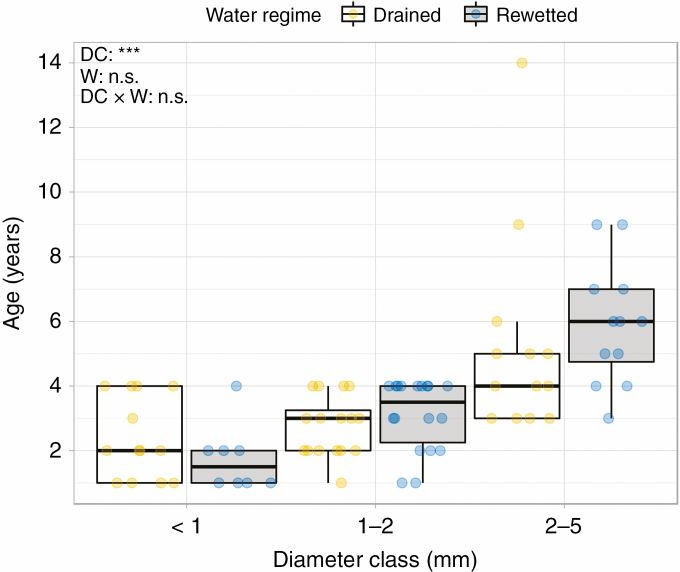
Age (years) of roots sampled at depth 10–20 cm for each diameter class (<1, 1–2 and 2–5 mm) in the rewetted and drained sites (*n* = 80). Asterisks (***) and n.s. in the upper left corner indicate significant (*P* < 0.001) and non-significant differences, respectively, between diameter classes (DC) and water regime (W, drained and rewetted).

Out of the 90 initial root samples for root age analysis, ten were discarded due to damage during preparation and/or uncertainties in the ring counting. In addition, measurements of the exact root diameter were only performed in 60 roots, since the bark was removed or damaged during section preparation and thus could not be accounted for. Root age generally increased with diameter class (*F* = 33.3, *P* < 0.001; Fig. 6), but did not differ significantly between the rewetted and drained sites (*F* = 0.0, *P* = 0.994). The actual measured diameter of roots (not diameter class) explained 44 % of the variance in root age for the drained site (*R*^2^ = 0.44, *F*_1,26_ = 20.7, *P* < 0.001; Supplementary Data Fig. S3), and 55 % of the variance in root age for the rewetted site (*R*^2^ = 0.55, *F*_1,30_ = 37.1, *P* < 0.001; Supplementary Data Fig. S3). The low *R*^2^ values are related to the relatively wide range of age (zero to four growth rings) in roots of smaller diameter classes (<1 and 1–2 mm) regardless of their measured diameter (Supplementary Data Fig. S3). Mean root age was 2.1 ± 0.3 years for the <1 mm class, 2.9 ± 0.2 years for the 1–2 mm class and 5.5 ± 0.5 years for the 2–5 mm diameter class.

## DISCUSSION

### Effects of water regime on root biomass distribution versus functional characteristics

*Alnus glutinosa* showed higher root biomass in the rewetted than in the drained site in almost the entire sampled soil profile, from 0 to 40 cm. The rewetted site is characterized by longer periods with high water table levels above the surface, while the drained site is characterized by longer periods in which the water table falls below the soil surface (Fig. 2A) leaving the rhizosphere exposed to oxygen. In addition, plant-available soil N concentration at the drained site was higher than at the rewetted site (Fig. 3A). High soil N concentrations are often found in degraded forest peatlands due to their high decomposition rates after aeration of the soil caused by drainage (Laiho, 1998; Nieminen *et al.*, 2017). When *A. glutinosa* is growing under waterlogged conditions in anaerobic soils, oxygen may enter the stems through enlarged lenticels, and is transported via aerenchyma (i.e. tissue containing enlarged gas spaces) to roots (McVean, 1956; Schröder, 1989*a*, *b*; Machacova *et al.*, 2013). This adaptation to water-saturated conditions enables the tree to transport oxygen into the otherwise anoxic rhizosphere, allowing root growth deep into waterlogged soils (Evans, 2004; Voesenek *et al.*, 2006; Colmer and Voesenek, 2009) and explains why root growth of *A. glutinosa* is not negatively affected by waterlogging. We were not able to identify aerenchyma in our samples of *A. glutinosa* roots because, as in many species with secondary growth (Philipson and Coutts, 1980; Wang and Cao, 2012), this tissue is found in the root cortex (Schweingruber *et al*., 2013), which was not properly preserved in our xylem-focused preparations. The ability of *Alnus* species to form symbiotic relationships with *Frankia* and to fix N is also an important feature that improves growth and facilitates nutrient uptake in N-poor conditions (Barea and Azcon-Aguilar, 1983; Orfanoudakis *et al.*, 2010). Previous studies on *A. glutinosa* found that high N concentrations (in the form of NO_3_^−^ or NH_4_^+^) reduce nodule initiation and growth as well as nitrogenase activity, since sufficient amounts of N are available for the trees and thus reduce the need for the symbiotic N-fixation (Bond *et al.*, 1954; Huss-Danell *et al.*, 1982; Gentili and Huss-Danell, 2003). Furthermore, a study on *Alnus maritima* showed that nodule growth and nitrogenase activity are inhibited in soils with low oxygen concentrations (Dawson, 1978; Kratsch and Graves, 2005). The increased root biomass in the rewetted site might be related to the lower oxygen concentrations under waterlogging, which might reduce nodule activity, forcing the tree to rely more on nutrient acquisition through roots. Since the rewetted site has lower N concentrations, more roots would be needed to fulfil the nutrient requirements. Additionally, the larger root biomass under rewetting could be linked to the need to oxidize the rhizosphere by releasing oxygen from the root tips to facilitate nutrient uptake in anoxic soil conditions (Wötzel, 1997).

The very fine root compartment (<1 mm) had the highest biomass compared with the 1–2 mm fine roots and the 2–5 mm coarse roots in both sites. Despite the larger biomass of the very fine roots <1 mm at the rewetted site, their functional characteristics did not differ between sites (although the small trend of higher SRA in the rewetted site could be related to a higher proportion of aerenchyma tissue in these roots; Fig. 5). Therefore, *A. glutinosa* seems to respond to rewetting by increasing biomass investments in very fine roots <1 mm rather than modifying their functional characteristics. Biomass of 1–2 mm fine roots was also significantly higher in the rewetted site than in the drained site, but that of the 2–5 mm coarse roots did not differ between water regimes (Fig. 4B, C). The SRA, however, was significantly higher in the drained site for these two diameter classes (Fig. 5). Therefore, while biomass of the very fine roots <1 mm was higher in the rewetted site, but showed almost no change in functional characteristics, we found the opposite pattern for the 2–5 mm coarse roots (no difference in root biomass between the two water regimes, but a significantly lower SRA in the rewetted site; Fig. 5). The large differences in SRA between diameter classes within the drained site, but not in the rewetted site, might be explained by the larger fluctuations in the water table in the drained site (Fig. 2A). These fluctuations may have large effects across the depth profile and as a result affect roots differently, since the distribution of diameter classes changes within this profile. The differences in SRA between the <1 mm and 1–2 mm roots suggest potential functional differences even between these small-diameter roots in *A. glutinosa* under fluctuating water table levels. Our findings indicate that in the drained site plants invested proportionally less resources in mass for roots of 1–5 mm (Supplementary Data Fig. S4A), while plants in the rewetted site invested proportionally more in mass (Supplementary Data Fig. S4B), resulting in a significantly higher SRA at the drained site. In fact, we would have expected to find lighter roots with higher SRA in the rewetted site due to a higher need for aerenchyma tissue under (seasonal) flooded conditions. Very fine roots <1 mm showed a tendency in this direction, but the observed opposite pattern for the fine (1–2 mm) and coarse (2–5 mm) roots is difficult to explain considering the higher aeration and the higher nutrient content at the drained site. Roots with higher SRA or SRL are not just expected to have higher resource acquisition efficiency, but also higher respiration rates and shorter lifespan, thus impacting soil C cycling (Makita *et al.*, 2012; McCormack *et al*., 2012; Bardgett *et al.*, 2014). Our findings indicate that rewetting not only supports the peatland function as C sink by enabling the system to sequester more C in form of root biomass, but also might lead to lower respiration rates of roots with a lower SRA in the rewetted state.

### Depth distribution and root age determine C input into the soil

Biomass distribution throughout the depth profile varies, mirroring the change in soil properties with soil depth (e.g. oxygen content, soil moisture, bulk density, temperature, nutrient availability or soil texture) (Schenk and Jackson, 2005). With increasing soil depth, root biomass decreased for very fine roots <1 mm (Fig. 4A), did not change for 1–2 mm fine roots (Fig. 4B) and increased for 2–5 mm coarse roots (Fig. 4C). Similar results were previously found for other temperate tree species from drier sites (i.e. *Acer saccharum*, *Fagus grandifolia* and *Betula alleghaniensis*), where roots <2 mm in diameter were more concentrated in shallow soil layers compared with 2–5 mm roots (Yanai *et al.*, 2008). Comparable results were also found for *Quercus serrata*, where the biomass of roots <0.5 and 0.5–1 mm, but not that of 1–2 mm roots, decreased with soil depth (Makita *et al.*, 2011). In our study, very fine roots <1 mm made up 51 % of the total root biomass, with the highest proportion in the shallow soil layers (0–10 and 10–20 cm). These results reflect previous accounts of the root system of *A. glutinosa*, with horizontal nutrient-absorbing roots growing at the surface (McVean, 1953, 1956), where higher oxygen and nutrient availability facilitate the uptake of nutrients (Gill and Burke, 2002). Our water table measurements show that the upper soil layers experienced the lowest number of water-saturated days (i.e. days when water was above the soil surface, so the rhizosphere could be considered as completely flooded) compared with the deeper soil layers (Supplementary Data Table S2). Accordingly, there were also longer periods with higher oxygen availability in the surface layers than in the lower soil depths in our study sites.

We found that root diameter explained a significant proportion of variance in root age (Supplementary Data Fig. S3) and very fine roots <1 mm were the youngest, on average 2.1 ± 0.3 years. Our age estimations of fine roots are in line with previous findings showing that the mean chronological age of fine roots in temperate and boreal forests usually ranges between 1 and 3 years (Solly *et al.*, 2018). Since these fine and young roots in our alder stands made up half of the total root biomass proportion and are mainly located in soil depths 0–20 cm, we can assume that turnover rates of roots and nutrients are higher in these shallow layers than in deeper soil layers, where the proportion of larger and older coarse roots (2–5 mm) is higher. This pattern coincides with previous findings that fine roots <0.5 mm and, in general, those near the surface have a more rapid turnover than 0.5–2 mm roots and deeper roots in a mature hardwood forest dominated by *Quercus* species (Joslin *et al.*, 2006). Furthermore, respiration rates of fine roots (≤2 mm) are much higher and more variable than those of larger-diameter roots of broad-leaved forests (Pregitzer *et al.*, 1998; Makita *et al.*, 2012). High root respiration is a major source of CO_2_ efflux from forests soils (Valentini *et al.*, 2000) and can account for up to 41% in afforested peatlands (Mäkiranta *et al.*, 2008). Thus, a higher biomass allocation to fine roots compared with larger roots could drive changes in soil C storage and nutrient cycling through greater C inputs into a more dynamic C compartment, and could counteract to some extent the increased C sink through biomass inputs with rewetting.

Our findings that larger-diameter roots (2–5 mm) are more abundant at deeper depths matches previous findings in broad-leaved temperate forests (Wells and Eissenstat, 2002; Makita *et al.*, 2011). Their longer lifespan (Wells and Eissenstat, 2002; Guo *et al.*, 2008) seems to compensate for the higher C investment per root area/length compared with smaller-diameter roots (Eissenstat and Yanai, 1997). The deeper soil layers are more likely to be at least seasonally water-saturated in these forest peatland ecosystems (Fig. 2A) and the low oxygen availability under anoxic soil conditions impedes decomposition of roots (Moore *et al.*, 2007). More C invested in larger, longer-living roots with slower turnover rates in combination with lower decomposition rates due to anoxic conditions favours a slower cycling of C in deeper soil layers.

### Conclusions

The highest proportion of roots up to 5 mm in *A. glutinosa* is made up of very fine roots of <1 mm. *Alnus glutinosa* increased investments in very fine roots <1 mm under rewetting rather than altering the root functional characteristics, while coarse roots of 2–5 mm showed the opposite response. These contrasting patterns in biomass versus functional traits under rewetting underline the differences between fine and coarse roots, not only in size, but also in their functional response to environmental changes. In addition, the large differences found in SRA between diameter classes within the drained site, which has larger water table fluctuations, emphasize the importance of the water regime with respect to root structure and function. The combination of root age determination and root biomass distribution in diameter classes and depths indicates higher rates of C turnover in shallow soil layers (<20 cm) in both sites, and higher below-ground C investments in the rewetted site. Rewetting of forest peatlands supports their function as a C sink by enhancing renewed C sequestration in the form of root biomass, which is another piece of evidence supporting peatland rewetting for climate change mitigation strategies.

## SUPPLEMENTARY DATA

Supplementary data are available online at https://academic.oup.com/aob and consist of the following. Table S1: data on the trees next to which soil cores were taken in the drained and rewetted alder stands. Table S2: abiotic parameters for the drained and rewetted alder stands. Table S3: results of Tukey’s HSD comparison of root biomass of *A. glutinosa*. Figure S1: gravimetric water content in the rewetted and drained alder forest stands. Figure S2: SRA across the depth profile. Figure S3: relationship between measured root diameter and age of roots sampled at depth 10–20 cm in the rewetted and drained alder forest stands. Figure S4: total root area in relation to total root mass of roots.

mcaa195_suppl_Supplementary_Materials
