# Supplementary material for: Root biomass and root traits of Alnus glutinosa show size-dependent and opposite patterns in a drained and a rewetted forest peatland
Source: Ann Bot. 2020 Sep 10;127(3):337–46. doi: 10.1093/aob/mcaa195 (PMC7872119; doi:10.1093/aob/mcaa195)
Supplement: mcaa195_suppl_Supplementary_Materials [file mcaa195_suppl_supplementary_materials.doc]

# Supplementary information

Table S1. Data on the trees next to which soil cores were taken in the drained and rewetted alder stands, including diameter at breast height (DBH in cm) and total height of the tree (m).

| hydrological  status | Soil core ID | DBH [cm] | Height [m] |
| --- | --- | --- | --- |
| drained | AD01 | 41.3 | 25.4 |
| drained | AD02 | 38.4 | 23.4 |
| drained | AD03 | 37 | 22.6 |
| drained | AD04 | 49.5 | 25 |
| drained | AD05 | 42 | 24.9 |
| rewetted | AW1* | 34.5 | 20.6 |
|  |  | 31.0 | 21.5 |
| rewetted | AW2 | 40.5 | 27.5 |
| rewetted | AW3 | 30.0 | 23.8 |
| rewetted | AW4* | 32.5 | 18.7 |
|  |  | 17.0 | 12.6 |
| rewetted | AW5 | 40.0 | 17.6 |
| *Asterisks indicate multi-stemmed trees, for which data on both stems are provided | | | |

Table S2. Abiotic parameters for the drained and rewetted alder stands. Annual temperature (°C), including air (2 m) and soil temperatures (5 cm, 15 cm and 50 cm) and annual precipitation sum (mm) were obtained from the same weather station locally installed between the two stands, due to the close proximity and shared climatic conditions between them. Water saturated days (WSD) are the counts of days when water table depth has risen above the measured soil depths. Water saturated days in the depth ‘air’ refer to days when water was above soil surface (0 cm), so the rhizosphere could be considered as completely flooded.

|  |  |  |  |  |  |  |  |  |  |
| --- | --- | --- | --- | --- | --- | --- | --- | --- | --- |
| hydrological status |  | depth |  | temperature |  | WSD |  |  | precipitation |
|  |  |  |  |  |  |  |  |  |  |
|  |  | [cm] |  | [°C] |  | [d] |  |  | [mm] |
|  |  |  |  |  |  |  |  |  |  |
|  |  |  |  |  |  |  |  |  |  |
| drained |  | air |  | 9.9 |  | 28 |  |  | 379.8 |
|  |  | 5 |  | 9.4 |  | 36 |  |  |  |
|  |  | 15 |  | 9.4 |  | 77 |  |  |  |
|  |  | 50 |  | 9.7 |  | 184 |  |  |  |
|  |  |  |  |  |  |  |  |  |  |
| rewetted |  | air |  | 9.9 |  | 165 |  |  |  |
|  |  | 5 |  | 9.7 |  | 168 |  |  |  |
|  |  | 15 |  | 9.8 |  | 175 |  |  |  |
|  |  | 50 |  | 10.2 |  | 193 |  |  |  |

Table S3. Results of the Tukey’s HSD comparison (R package *emmeans*) of root biomass of *A. glutinosa* for the three diameter classes (<1 mm, 1-2 mm and 2-5 mm) between the five depths (0-10, 10-20, 20-30, 30-40 and 40-50 cm) including estimated marginal means (emmean) and standard error (SE). Effects were considered significant at p<0.05 and are indicated in bold.

|  |  |  |  |  |  |  |  |  |  |  |
| --- | --- | --- | --- | --- | --- | --- | --- | --- | --- | --- |
|  |  | emmean | SE |  | df |  | depth in cm | | | |
|  |  |  |  |  |  |  | 10-20 | 20-30 | 30-40 | 40-50 |
|  |  |  |  |  |  |  |  |  |  |  |
| *<1 mm* |  |  |  |  |  |  |  |  |  |  |
| 0-10 |  | 4.75 | 0.28 |  | 40 |  | 0.694 | 0.359 | **<0.001** | **<0.001** |
| 10-20 |  | 5.15 | 0.28 |  | 40 |  | - | **0.019** | **<0.001** | **<0.001** |
| 20-30 |  | 4.19 | 0.28 |  | 40 |  | - | - | **0.004** | 0.058 |
| 30-40 |  | 3.07 | 0.28 |  | 40 |  | - | - | - | 0.932 |
| 40-50 |  | 3.32 | 0.29 |  | 45 |  | - | - | - | - |
|  |  |  |  |  |  |  |  |  |  |  |
| *1-2 mm* |  |  |  |  |  |  |  |  |  |  |
| 0-10 |  | 3.49 | 0.28 |  | 40 |  | 1.000 | 0.856 | 0.127 | 0.769 |
| 10-20 |  | 3.54 | 0.28 |  | 40 |  | - | 0.780 | 0.091 | 0.682 |
| 20-30 |  | 3.18 | 0.28 |  | 40 |  | - | - | 0.615 | 1.000 |
| 30-40 |  | 2.73 | 0.29 |  | 45 |  | - | - | - | 0.763 |
| 40-50 |  | 3.12 | 0.29 |  | 45 |  | - | - | - | - |
|  |  |  |  |  |  |  |  |  |  |  |
| *2-5 mm* |  |  |  |  |  |  |  |  |  |  |
| 0-10 |  | 4.18 | 0.29 |  | 45 |  | 1.000 | 0.925 | 0.923 | 0.227 |
| 10-20 |  | 4.16 | 0.28 |  | 40 |  | - | 0.938 | 0.936 | 0.173 |
| 20-30 |  | 3.91 | 0.29 |  | 45 |  | - | - | 1.000 | **0.034** |
| 30-40 |  | 3.90 | 0.30 |  | 53 |  | - | - | - | **0.042** |
| 40-50 |  | 4.87 | 0.29 |  | 45 |  | - | - | - | - |
|  |  |  |  |  |  |  |  |  |  |  |
|  |  |  |  |  |  |  |  |  |  |  |


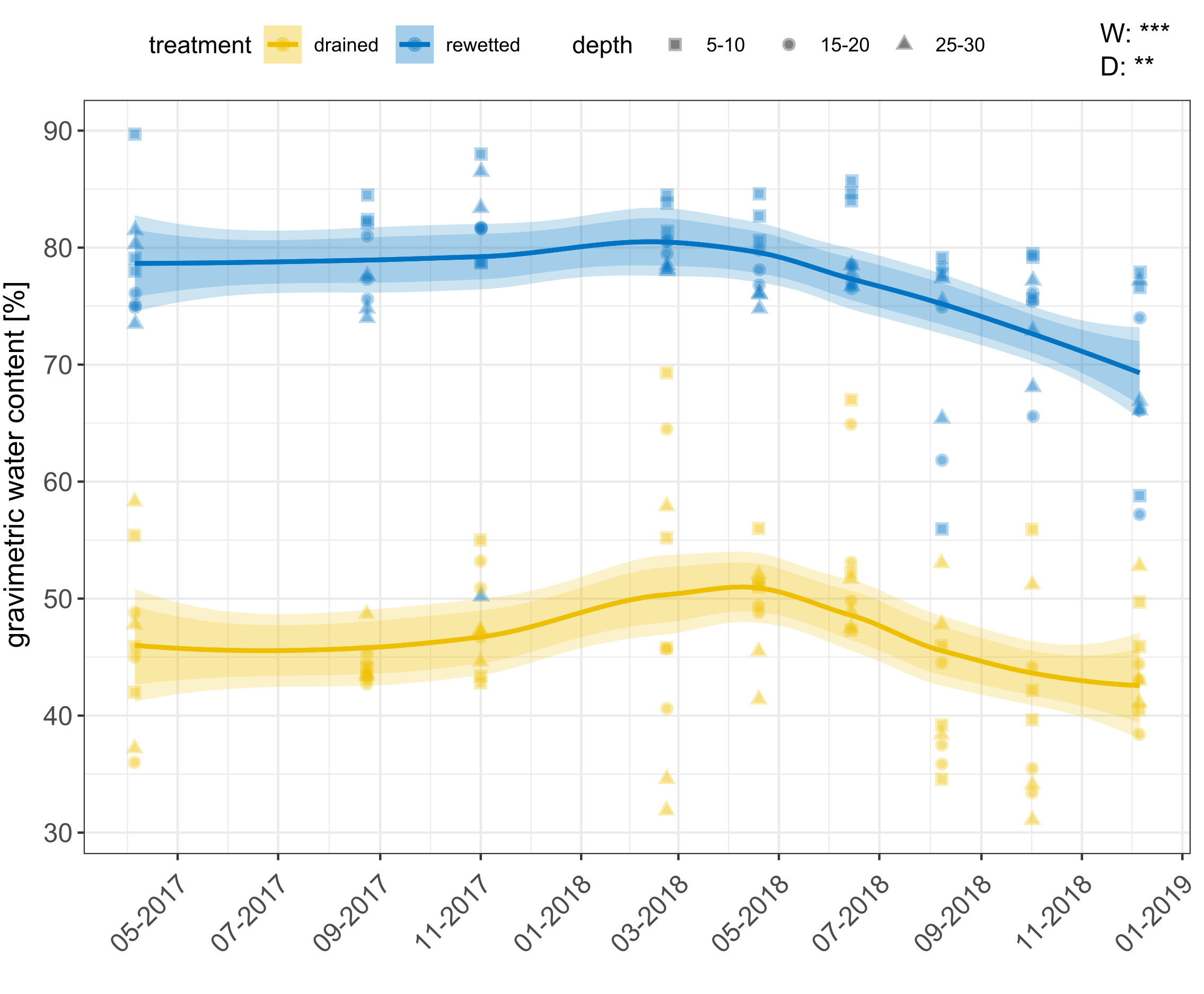


Fig. S1. Gravimetric water content (%) in the rewetted (blue line/symbols) and drained (yellow line/symbols) alder forest stands (N=162), measured in the soil depths 0-5 cm (squares), 15-20 cm (circles), and 25-30 cm (triangles). Asterisks indicate significant differences between fixed-effect variables (*** p<0.001, ** p<0.01). W = water regime; D = depth. Bands show 83 and 95% confidence interval.


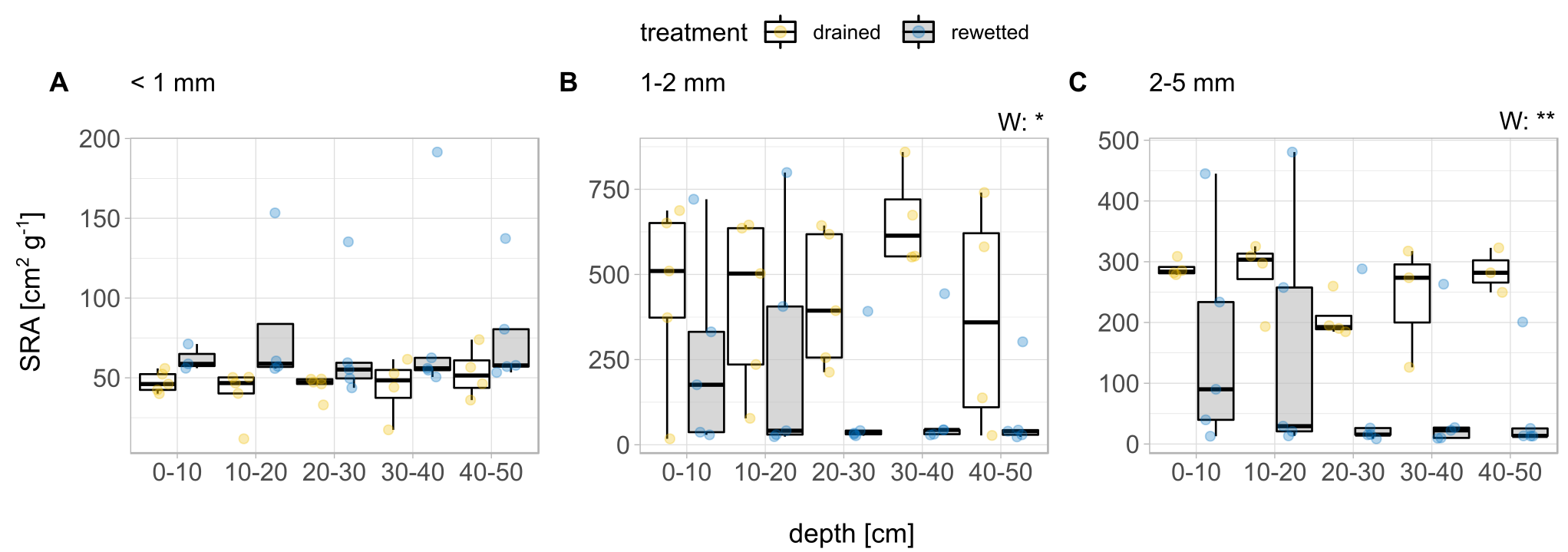
Fig. S2. Specific root area (SRA, cm² g^-1^) across the depth profile (0-10, 10-20, 20-30, 30-40 and 40-50 cm) for the diameter classes (A) <1 mm, (B) 1-2 mm, and (C) 2-5 mm in the rewetted (blue symbols, grey boxes) and drained (yellow symbols, white boxes) alder forest stands. Asterisks indicate significant differences between fixed-effect variables (** p<0.01, * p<0.05). Non-significant differences are not stated. W = water regime (drained and rewetted).


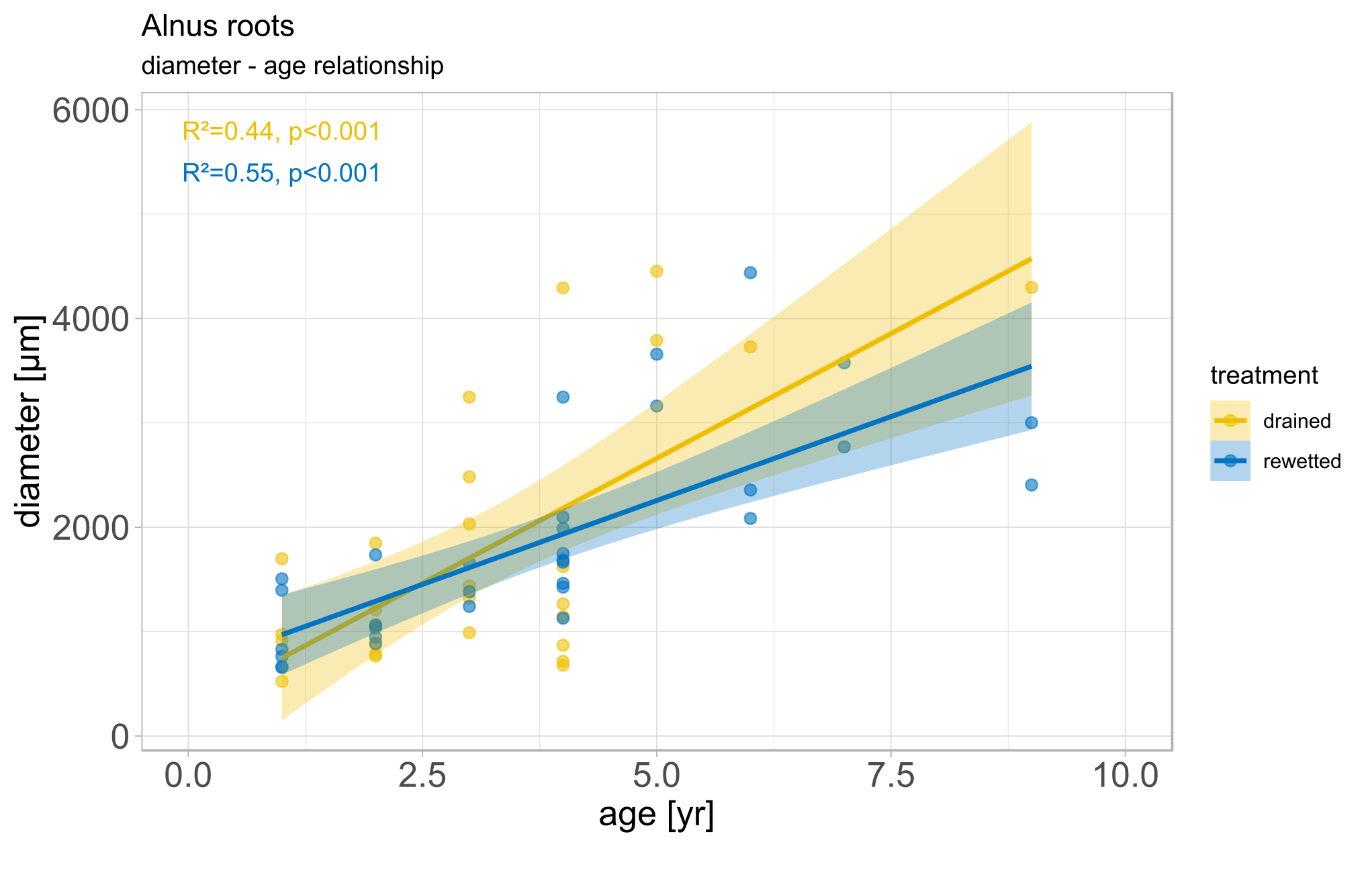
Fig. S3. Relationship between the measured root diameter (µm) and root age (years) of roots sampled at the depth 10-20 cm in the rewetted (blue line/symbols) and drained (yellow line/symbols) alder forest stands (N=60). Bands show 95% confidence interval.


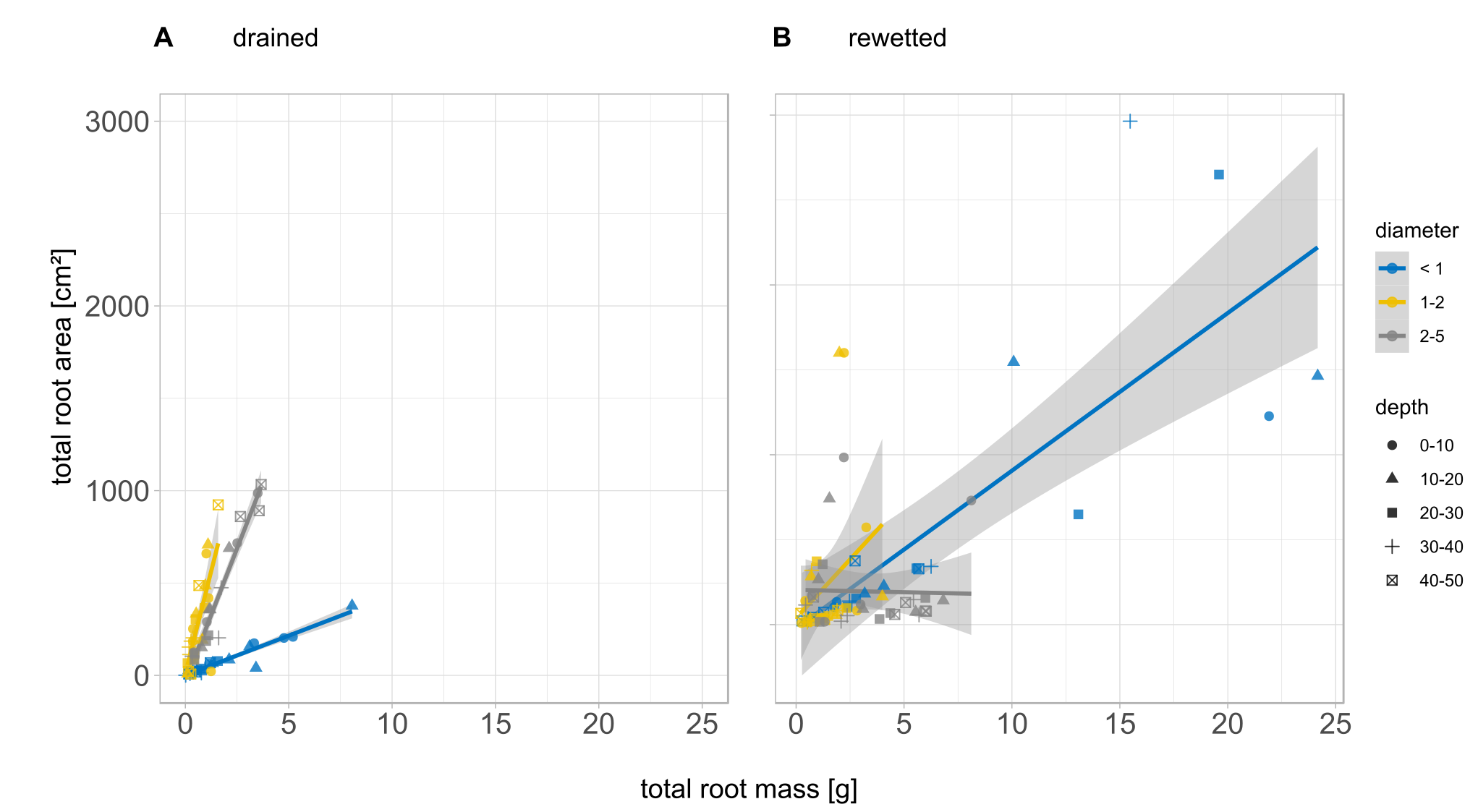
Fig. S4. Total root area (cm²) in relation to total root mass (g) of roots of the diameter classes <1 mm (blue), 1-2 mm (yellow), and 2-5 mm (grey) in the soil depths 0-10 cm (circles), 10-20 cm (triangles), 20-30 cm (squares), 30-40 cm (crosses) and 40-50 cm (crossed squares) in the drained (A) and rewetted (B) alder forest stands. Grey bands show 95% confidence interval.
